# Comparison of catheter wound infusion, intrathecal morphine, and intravenous analgesia for postoperative pain management in open liver resection: randomized clinical trial

**DOI:** 10.1093/bjsopen/zraf074

**Published:** 2025-07-15

**Authors:** Damien Rousseleau, Barthélémy Plane, Julien Labreuche, Adeline Pierache, Younes El Amine, Sabine Ethgen, Jean-Michel Wattier, Cédric Cirenei, Emmanuel Boleslawski, Gilles Lebuffe

**Affiliations:** Department of Anesthesiology and Critical Care, Hospital Claude Huriez, Lille University Hospital, F59000-Lille, France; ULR 7365-GRITA-Groupe de Recherche sur les formes Injectables et les Technologies Associées, Lille University, F59000-Lille, France; Department of Anesthesiology and Critical Care, Hospital Claude Huriez, Lille University Hospital, F59000-Lille, France; Department of Biostatistics, CHU Lille, F59000-Lille, France; Department of Biostatistics, CHU Lille, F59000-Lille, France; Department of Anesthesiology and Critical Care, Hospital Claude Huriez, Lille University Hospital, F59000-Lille, France; Department of Anesthesiology and Critical Care, Hospital Claude Huriez, Lille University Hospital, F59000-Lille, France; Department of Anesthesiology and Critical Care, Hospital Claude Huriez, Lille University Hospital, F59000-Lille, France; Department of Anesthesiology and Critical Care, Hospital Claude Huriez, Lille University Hospital, F59000-Lille, France; ULR 2694—METRICS: Evaluation des Technologies de santé et des Pratiques médicales, Lille University, Lille University Hospital, F59000-Lille, France; Department of Digestive Surgery and Transplantation, Lille University Medical Hospital, University of Lille Nord de France, F59000-Lille, France; Department of Anesthesiology and Critical Care, Hospital Claude Huriez, Lille University Hospital, F59000-Lille, France; ULR 7365-GRITA-Groupe de Recherche sur les formes Injectables et les Technologies Associées, Lille University, F59000-Lille, France

## Abstract

**Background:**

Pain relief is an important aspect of recovery after open liver resection. This randomized open-label single-centre trial assessed the efficacy of intravenous (i.v.) analgesia alone or in combination with catheter wound infusion (CWI) or intrathecal morphine (ITM) after open liver resection.

**Methods:**

Adult patients undergoing open liver resection were randomly assigned to receive either i.v. analgesia alone or in combination with ITM or CWI. In this study, i.v. analgesia consisted of systematic i.v. paracetamol and i.v. morphine via a patient-controlled analgesia pump, with i.v. nefopam as rescue analgesia for a Numeric Rating Scale (NRS) score > 4. The primary outcome was cumulative morphine dose at 24 hours (h). Secondary outcomes included pain intensity, cumulative opioid use at 48 and 72 h, and postoperative complications.

**Results:**

In all, 186 patients were included in the study (62 patients in each group). The median 24-h morphine dose was 14 (interquartile range (i.q.r.) 6–25) mg in the i.v. analgesia group, 14 (i.q.r. 7–23) mg in the CWI group, and 7 (i.q.r. 3–15) mg in the ITM group. ITM significantly reduced morphine use compared with i.v. analgesia alone (mean difference on log-transformed values 0.57; 95% confidence interval 0.21 to 0.93; Bonferroni-adjusted *P* = 0.002) and lowered pain scores during the first 12 h. No significant differences were observed between the CWI and i.v. analgesia groups. By 72 h, cumulative opioid use was similar across all groups. Adverse events and postoperative complications were comparable across the three groups.

**Conclusion:**

ITM reduced the cumulative morphine dose and pain intensity in the first 24 h after liver resection, providing a valuable option for postoperative analgesia.

**Registration number:**

NCT03238430 (http://www.clinicaltrials.gov).

## Teaser

This study compared the effectiveness of intravenous (i.v.) analgesia alone *versus* i.v. analgesia in combination with intrathecal morphine (ITM) or catheter wound infusion (CWI) for postoperative pain management in patients undergoing open liver resection. ITM significantly reduced the cumulative morphine dose and pain intensity in the first 24 h compared with i.v. analgesia alone or CWI. These findings suggest that ITM provides superior early postoperative pain relief in liver surgery.

## Introduction

Effective postoperative pain management is essential for enhancing recovery and reducing hospital stays in patients undergoing open liver resection. An optimal therapeutic strategy for postoperative pain should aim to achieve zero to low pain levels, defined as a Numerical Rating Scale (NRS) score < 3, both at rest and during mobilization. It should also minimize morphine use and promote enhanced postoperative rehabilitation. According to the most recent PROSPECT recommendations^1^, thoracic epidural analgesia (TEA) has been shown to be an effective way to treat pain within the first 24 hours (h) after surgery. Due to concerns about hypotension and reduced mobility, which may hinder the recovery process, the most recent Enhanced Recovery After Surgery (ERAS) Society guidelines^2^ recommend against the routine use of TEA. This recommendation aligns with the findings of Burchard *et al*.^3^, who demonstrated that early adherence to ERAS protocols was a predictor of shorter hospital stays.

When TEA is not used, the ERAS Society guidelines recommend alternative strategies, such as plane blocks or wound infiltration, supported by several meta-analyses^4–6^. In contrast, the PROSPECT guidelines^1^ only recommend plane block. Another option endorsed by the ERAS Society guidelines is the use of intrathecal morphine (ITM) for postoperative analgesia, which is not supported by the PROSPECT guidelines due to a lack of evidence.

Despite these guidelines, no studies have directly compared these various analgesic approaches in the context of open liver resection. Consequently, the optimal strategy for postoperative pain management within an ERAS protocol remains unclear. The aim of the present study was to fill that gap by comparing the effects of intravenous (i.v.) analgesia alone or in combination with either postoperative continuous wound infiltration (CWI) or preoperative ITM in patients undergoing open liver resection.

## Methods

### Study design

This prospective randomized parallel open-label clinical trial followed the CONSORT guidelines^7^. The study was conducted to evaluate i.v. analgesia alone or in combination with either postoperative CWI or preoperative ITM in patients undergoing open liver surgery at the Department of Transplant and Liver Surgery, University Hospital of Lille, France. Participants were randomly (without stratification) assigned in a 1 : 1 : 1 ratio to either ITM, CWI, or i.v. analgesia alone. The primary outcome was cumulative morphine dose (mg) in the first 24 h after the surgery.

### Study population

To be eligible for inclusion, patients had to be aged 18–75 years, have an American Society of Anesthesiologists (ASA) grader of I–III, have provided signed informed consent, and to have undergone liver resection by laparotomy. Exclusion criteria were contraindications to ITM, a history of chronic pain or drug addiction, chronic preoperative use of opioids or non-opioid analgesics, and allergies to any analgesics administered in the study.

The trial was approved by the Research Ethics Committee of Paris North West (reference no. 201400331728) and validated by the Clinical Research Federation of the Lille University Hospital. Written informed consent was obtained from all enrolled patients. The study was preregistered at ClinicalTrials.gov (NCT03238430), and patients were enrolled between May 2015 and December 2020.

### Randomization and study drug administration

Randomization was performed using sealed envelopes containing a computer-generated sequence provided by an independent statistician, with block sizes of six. Group allocation was disclosed to patients, who were informed about the protocol during the preoperative anaesthesia consultation. Signed consent was collected the day before surgery.

The i.v. analgesia regimen consisted of the systematic administration of paracetamol (1.0 g every 6 h) and postoperative i.v. morphine delivered via a patient-controlled analgesia (PCA) pump for 72 h. The PCA pump contained a solution of 50 ml morphine (1 mg/ml) and droperidol (0.05 mg/ml), with a 1-mg bolus dose and a 5-minute lockout interval. Nefopam (20 mg every 4 h) and tramadol (50 mg every 6 h) were administered as second- and third-line analgesics, respectively, if pain persisted with an NRS score > 4, despite the use of paracetamol and morphine.

For patients in the ITM group, the anaesthetist administered a single intrathecal dose of morphine (0.3 mg in 4 ml of 0.9% sodium chloride) via lumbar puncture at L3/L4 or L4/L5 with a 27-gauge needle before the induction of anaesthesia. In the CWI group, a multiperforated catheter (Profils Paincath P500-30QR) was placed by a senior surgeon between the obliquus externus and internus abdominis muscles during incision closure. Ropivacaine (2 mg/ml) was administered via the catheter, starting with a 20-mg bolus followed by a continuous infusion at rate of 8 ml/h. The catheter was removed 48 h after surgery. The complete anaesthetic management protocol is provided in the *Supplementary material*.

### Data collection and endpoint measures

The primary endpoint was the cumulative morphine dose at 24 h after surgery. Secondary endpoints included the cumulative morphine dose at 48 and 72 h, pain intensity (NRS) at rest and during mobilization, the incidence of postoperative nausea and vomiting (PONV), anti-emetic use, time to return of bowel function, and the length of intensive care unit (ICU) and hospital stays.

Cumulative morphine dose was recorded at 24, 48, and 72 h after surgery. Pain intensity was measured at 3, 6, 12, 24, 48, and 72 h using the NRS. PONV, time to first bowel movement, and the time of resumption of feeding were recorded, as was the use of ondansetron. Postoperative complications, the length of ICU stay, and the length of the total hospital stay were also noted.

### Statistical analysis

The sample size of 186 patients was calculated using a two-sided *t* test at the 0.025 significance level (to take into account the two comparisons between experimental arms and a control arm), assuming a 30% reduction in 24-h morphine consumption in each experimental arm compared with the control (i.v. analgesia alone) arm, with a mean(standard deviation) 24-h morphine consumption of 40(20) mg in the control arm and considering a 10% rate of attrition. A detailed statistical plan is provided in the *Supplementary material*. All analyses were performed on all randomized patients in their original randomization groups regardless of protocol deviations (according to an intention-to-treat (ITT) basis). The cumulative morphine dose at 24 h (primary endpoint), as well as 48 and 72 h, after surgery in each experimental arm (CWI and ITM) was compared to the control arm using one-way analysis of variance (ANOVA), with log transformation (values + 1) applied to meet normality assumptions. Secondary quantitative outcomes were compared between each experimental and the control arms using Dunn’s test^8^, and secondary binary outcomes were compared using χ^2^ tests. Finally, the pain intensity at rest and on coughing or mobilization assessed at six postoperative time points (3, 6, 12, 24, 48 and 72 h) was compared between each experimental and the control arms using linear mixed models (an unstructured covariance pattern model to account for the correlation between repeated measures within the same patients) with baseline values, times, analgesic group, and the interaction between times and analgesic group as fixed effects.

All statistical tests were two-sided, and *P* < 0.025 was considered statistically. All confidence intervals were two-sided at a 95% confidence level, and should not be used for hypothesis testing. Data were analysed using SAS (release 9.4) and R (release 3.6.2).

## Results

**Fig. 1:**
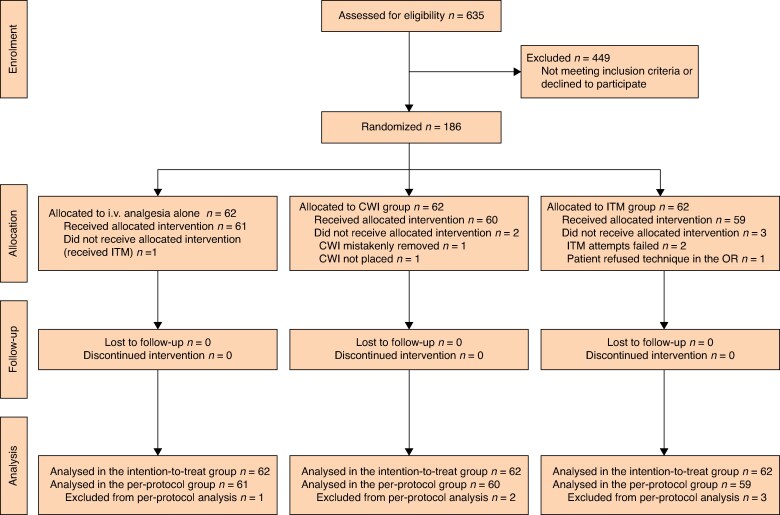
CONSORT flow diagram^7^ i.v. intravenous; CWI, catheter wound infiltration in addition to i.v. analgesia; ITM, intrathecal morphine in addition to i.v. analgesia.

In all, 186 patients were randomized into three groups (62 patients per group), and all were analysed on an ITT basis. Six patients were excluded from the per-protocol analysis of the primary endpoint due to non-compliance with the anaesthetic protocol (*Fig. 1*). Of these patients, one in the i.v. analgesic alone group received ITM. In the CWI group, one patient experienced accidental catheter removal and catheter insertion was missed in the other. In the ITM group, two patients did not receive ITM due to procedural failure, and one patient refused the injection.

Baseline characteristics were well balanced across the three groups (*Table 1*). Most patients were male, and the primary indications for liver resection were liver metastasis from colorectal cancer and biliary carcinoma. More than half the liver resections in each group involved major resections (more than three segments). Intraoperative variables, including procedure duration, sufentanil dose, blood loss, and fluid administration, were similar across the three groups.

**Table 1:** Baseline characteristics of patients in the i.v. analgesia alone, CWI, and ITM groups separately

	I.v. analgesia (*n* = 62)	CWI (*n* = 62)	ITM (*n* = 62)
**Sex**			
Male	46 (74%)	45 (73%)	47 (76%)
Female	16 (26%)	17 (27%)	14 (24%)
Age (years), mean(s.d.)	63.3(11.5)	64.8(10.4)	64.7(9.8)
BMI (kg/m^2^), mean(s.d.)	26.5(4.6)	26.6(5.1)	26.7(4.9)
**ASA grade**			
I	5 (8.1%)	6 (9.7%)	5 (8.1%)
II	40 (64.5%)	38 (61.3%)	38 (61.3%)
≥III	17 (27.4%)	18 (29.0%)	19 (30.6%)
**Indication for resection**			
Colorectal metastasis	33 (53%)	26 (42%)	34 (55%)
Primary malignancies	8 (13%)	19 (31%)	13 (21%)
Biliary carcinoma	19 (31%)	15 (24%)	11 (18%)
Benign tumours	2 (3%)	2 (3%)	4 (7%)
Major liver resection*	33 (53%)	39 (63%)	42 (68%)
Total blood loss (ml), median (i.q.r.)	500 (200–700)	500 (300–750)	500 (300–800)
Total volume intraoperative fluid administered (l), mean(s.d.)	2.9(1.0)	3.2(1.6)	3.0(1.0)
Total duration of operation (min), mean(s.d.)	328.6(114.2)	339.6(141.8)	316(116.4)

^*^Values are *n* (%) unless otherwise stated. Major liver resections correspond to three or more segments. i.v., intravenous; CWI, continuous wound infusion in addition to i.v. analgesia; ITM, intrathecal morphine in addition to i.v. analgesia; BMI, body mass index; ASA, American Society of Anesthesiologists.

**Fig. 2:**
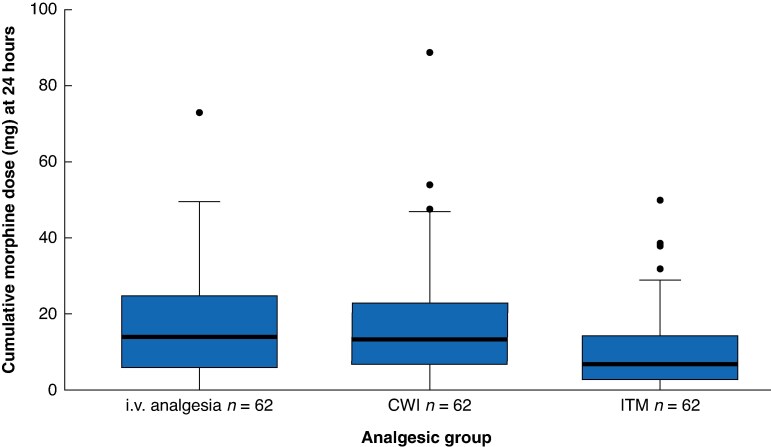
Distribution (Tukey’s box plot) of cumulative morphine doses at 24 hours (primary endpoint) according to analgesic group Patients received intravenous (i.v.) analgesia alone or i.v. analgesic combined with either continuous wound infusion of local anaesthetic via a multiperforated catheter (CWI) or intrathecal morphine (ITM). Boxes show the interquartile range, with the median indicated by the horizontal line. Whiskers indicate values outside the lower and upper quartiles with a length equal to 1.5× the interquartile range.

**Fig. 3:**
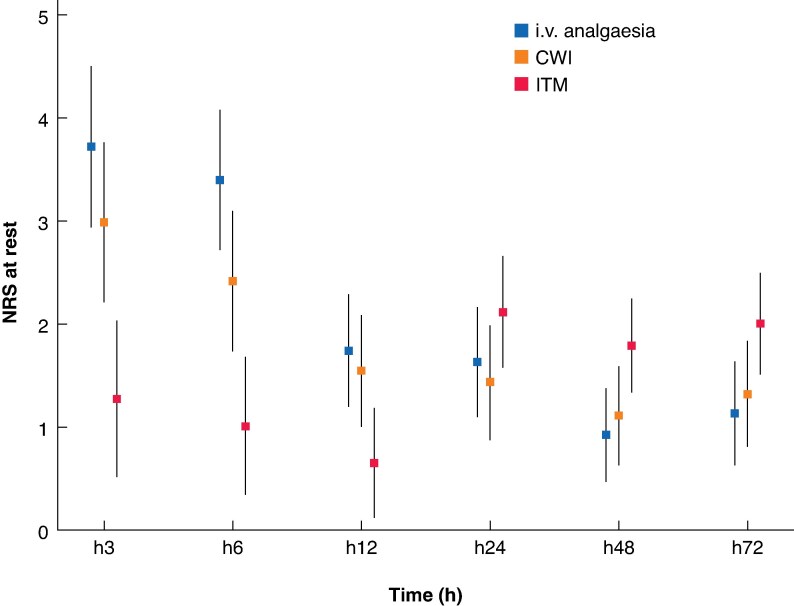
Mean NRS scores at rest over time in patients who underwent laparoscopic liver surgery according to analgesic group Patients received intravenous (i.v.) analgesia alone or i.v. analgesia combined with either continuous wound infusion of a local anaesthetic via a multiperforated catheter (CWI) or intrathecal morphine (ITM). Error bars indicate 95% confidence intervals. NRS, numerical rating scale; h, hours.

The cumulative morphine dose at 24 h was significantly lower in patients receiving ITM than in those receiving i.v. analgesia alone, with a median of 7 (interquartile range (i.q.r.) 3–15) mg *versus* 14 (i.q.r. 6–25) mg, and a mean between-group difference in log-transformed values of 0.57 (95% confidence interval (c.i.) 0.21 to 0.93; *P* = 0.002) (*Fig. 2*; *Table 2*). Similar results were found after adjustment for complexity of resection (*Table S1*) and in per-protocol analysis excluding the six patients without the allocated analgesic protocol (data not shown). No significant difference was found between patients receiving CWI and those receiving i.v. analgesia alone (mean between-group difference on log-transformed values 0.02; 95% c.i. −0.33 to 0.38). Morphine requirements became similar between each of the experimental groups and the control group from 48 h onwards. The median total morphine dose at 72 h remained low, not exceeding 27 mg (*Table 2*). Pain intensity at rest and during mobilization was lower in the ITM group than in the i.v. analgesia group during the first 12 h after surgery. At rest, the mean(standard deviation) numerical pain scores in the ITM group at 3, 6, and 12 h were 1.2(1.9), 1.0(2.0), and 0.7(1.4), respectively. In comparison, numerical pain scores at 3, 6, and 12 h were 2.9(2.7), 2.4(2.6), and 1.5(2.1), respectively, in the CWI group and 3.6(3.2), 3.4(2.8), and 1.7(2.2), respectively, in the i.v. analgesia group (*Fig. 3*; *P* < 0.001 at all time points for ITM *versus* i.v. analgesia). Pain scores in the ITM group were also significantly higher than in the i.v. analgesia group at both 48 h (*P* = 0.009) and 72 h (*P* = 0.016).

**Table 2:** Total morphine doses over the 72-h period after surgery, with comparisons between patients in the i.v. analgesia alone, CWI, and ITM groups

	i.v. analgesia (*n* = 62)	CWI (*n* = 62)	ITM (*n* = 62)	Effect size: control *versus* CWI	*P*_1_	Effect size: control *versus* ITM	*P*_2_
**Cumulative morphine dose (mg)**							
At 24 h (primary endpoint)							
Median (i.q.r.)	14 (625)	14 (7–23)	7 (3–15)				
Mean (log_e_)^†^	2.56 (2.31, 2.81)	2.53 (2.28, 2.78)	1.99 (1.73, 2.24)	0.02 (−0.33, 0.38)	0.890	0.57 (0.21, 0.93)	0.002*
At 48 h							
Median (i.q.r.)	20 (11–35)	20 (11–32)	18 (7–30)				
Mean (log_e_)^†^	2.95 (2.70, 3.20)	2.92 (2.67, 3.16)	2.74 (2.49, 2.99)	0.03 (−0.32, 0.38)	00.850	0.21 (−0.14, 0.56)	0.240
At 72 h							
Median (i.q.r.)	25 (15–53)	25 (17–46)	27 (13–50)				
Mean (log_e_)^†^	3.23 (2.96, 3.49)	3.18 (2.92, 3.44)	3.22 (2.96, 3.48)	0.05 (−0.32, 0.41)	0.800	0.01 (−0.36, 0.38)	0.970

^†^Values in parentheses are 95% confidence intervals. *P*_1_ and *P*_2_ are *P* values for comparison with the i.v. analgesia alone group, calculated after log_e_ + 1 transformation of cumulative morphine consumption data. The widths of the confidence intervals were not adjusted for multiple comparisons and should not be used for hypothesis testing. Effect sizes are for mean differences between the i.v. analgesia alone and the CWI and ITM groups (log_e_(cumulative morphine dose + 1) values). I.v., intravenous; CWI, continuous wound infusion in addition to i.v. analgesia; ITM, intrathecal morphine in addition to i.v. analgesia; i.q.r., interquartile range; h, hours. *Statistically significant at a two-sided significance level of 0.025 (Bonferroni adjusted significance level for the two experimental arm comparisons with the control arm).

**Fig. 4:**
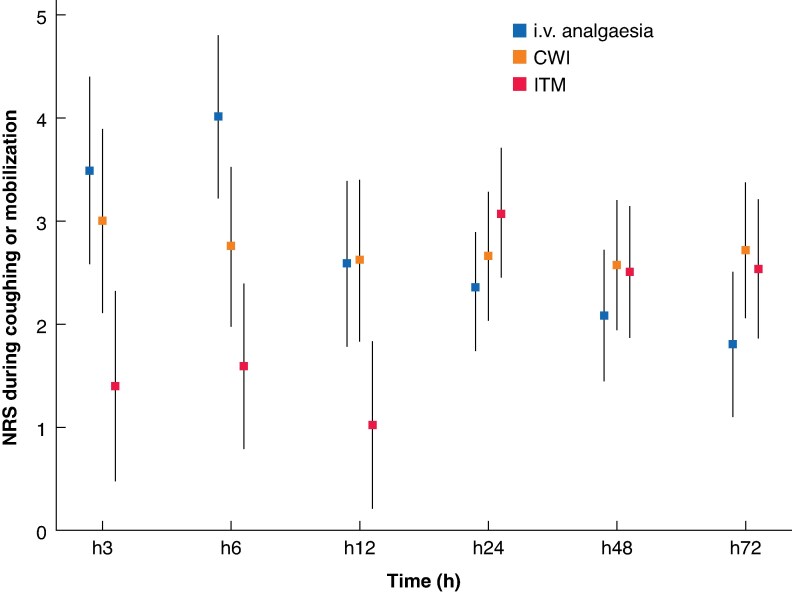
Mean NRS scores during coughing or mobilization over time in patients who underwent laparoscopic liver surgery according to analgesic group Patients received intravenous (i.v.) analgesia alone or i.v. analgesia combined with either continuous wound infusion of local anaesthetic via a multiperforated catheter (CWI) or intrathecal morphine (ITM group). Error bars indicate 95% confidence intervals. NRS, numerical rating scale; h, hours.

For pain during mobilization or coughing, the ITM group again had significantly lower scores in the first 12 h (mean(standard deviation) scores at 3, 6, and 12 h of 1.4(2.5), 1.7(2.5), and 1.0(1.9), respectively). In the CWI group, the scores at 3, 6, and 12 h were 3.6(3.4), 3.3(2.8), and 3.2(2.8), respectively, whereas in the i.v. analgesic group they were 4.4(3.4), 4.9(3.0), and 2.9(3.0), respectively (*Fig. 4*; *P* < 0.001 for ITM *versus* i.v. analgesia). From 24 h after surgery, no significant intergroup differences in pain intensity during mobilization were observed.

Supplementary analgesic consumption at 72 h was higher in the i.v. analgesia group (median nefopam dose 140 (i.q.r. 40–180) mg) than in the CWI (median nefopam dose 30 (i.q.r. 0–120) mg; *P* = 0.002) and ITM (median nefopam dose 60 (i.q.r. 0–120) mg; *P* = 0.0025) groups. Tramadol use did not differ significantly between groups, with median doses of 0 mg in all groups.

There were no significant differences in the ICU or hospital length of stays among groups (*Table 3*). In addition, PONV, ondansetron use, time to first bowel movement, time to resumption of feeding, pruritus, and respiratory depression were comparable across groups. Postoperative morbidity was similar across groups, affecting 18 patients (29%) in the i.v. analgesia group, 22 patients (36%) in the CWI group (*P* = 0.44), and 23 patients (37%) in the ITM group (Bonferroni-adjusted *P* = 0.34). Two patients in the CWI group developed wound abscesses that required catheter removal, and one patient had to have their catheter removed on the second postoperative day due to local anaesthetic leakage.

**Table 3:** Intergroup comparisons for length of hospital stay, morphine-related side effects, and criteria for return to independent feeding

	i.v. analgesia (*n* = 62)	CWI (*n* = 62)	ITM (*n* = 62)	Effect size: control *versus* CWI*	*P*_1_	Effect size: control *versus* ITM*	*P*_2_
Length of hospital stay (days), median (i.q.r.)	8 (7–12)	9 (7–13)	8 (7–11)	8.6 (−27.9, 45.0)	0.32	−6.5 (−42.6, 29.6)	0.37
Length of ICU stay (days), median (i.q.r.)	4 (2–6)	3 (2–5)	4 (3–6)	−6.5 (−42.9, 30.0)	0.36	5.2 (−30.9, 41.3)	0.39
Postoperative nausea and vomiting	11 (18%)	6 (9.7)	11 (18%)	1.8 (0.7, 4.6)	0.19	1.0 (0.5, 2.1)	0.97
Ondansetron dose at 72 h (mg), median (i.q.r.)	4 (4–7)	4 (4–8)	8 (4–8)	24.5 (−65.9, 114.9)	0.29	36.0 (−50.3, 122.3)	0.21
Time to first gas (days), median (i.q.r.)	2 (2–3)	2 (1–3)	2 (2–3)	−37.5 (−73.6, −1.4)	0.11	−23.5 (−59.8, 12.7)	0.065
Time to first bowel movement (days), median (i.q.r.)	4 (3–5)	4 (3–5)	4 (3–5)	−1.6 (−38.2, 34.9)	0.47	−0.7 (−37.4, 36.0)	0.49
Time to resumption of normal feeding (days), median (i.q.r.)	4 (3–5)	4 (3–6)	4 (3–5)	14.5 (−21.7, 50.6)	0.21	14.7 (−21.6, 51.0)	0.22
Pruritus	0 (0%)	0 (0%)	0 (0%)		ND		ND
Respiratory depression	2 (3%)	0 (0%)	2 (3%)		ND		ND

^*^Values in parentheses are 95% confidence intervals. Effect sizes are for standardized mean differences calculated on rank-transformed data, except for postoperative nausea and vomiting, for which relative risk is reported. The widths of the confidence intervals were not adjusted for multiple comparisons and should not be used for hypothesis testing. *P*_1_ and *P*_2_ are *P* values for comparisons with the i.v. analgesia alone group. i.v., intravenous; CWI, continuous wound infusion in addition to i.v. analgesia; ITM, intrathecal morphine in addition to i.v. analgesia; i.q.r., interquartile range; ICU, intensive care unit; ND, not done (for categorical variables with frequency of >5 patients).

## Discussion

This study aimed to compare the analgesic efficacy of an i.v. analgesic regimen combined with either CWI or ITM against i.v. analgesia alone in patients undergoing open liver resection. The results of this study demonstrate that ITM significantly reduced morphine consumption by half in the first 24 h after surgery compared with the CWI group and the i.v. analgesia alone groups. Pain scores were also significantly lower in the ITM group during the first 12 h after surgery, both at rest and during mobilization. This analgesic effect was transient, with no significant difference in pain or opioid use beyond 24 h.

CWI did not demonstrate superior pain management compared with the i.v. analgesic strategy. The findings of the present study contradict those of the latest meta-analysis by Sadik *et al.*^6^, which reported a benefit of CWI. The discrepancy with this systematic review can mostly be attributed to the lack of baseline analgesia in four of the six included studies. Among the studies that incorporated baseline analgesia, Dalmau *et al*.^9^ (baseline analgesia: paracetamol and non-steroidal anti-inflammatory drugs (NSAIDs) twice daily) demonstrated reduced pain intensity at 6 h after surgery, similar to the findings of the present study. However, this did not result in decreased morphine use during the first 48 h after surgery. In contrast, Karanicolas *et al.*^10^ (baseline analgesia: NSAIDs alone) reported reduced morphine use within the first 48 h after surgery, although no differences were observed during the first 24 h, when peak pain intensity typically occurs. In the study of Karanicolas *et al.*^10^, the median cumulative morphine dose was reported to be 40 (i.q.r. 25–66) mg, compared with 25 (range 20–29) mg in the study of Dalmau *et al*.^9^ and 20 mg (i.q.r. 11–35) in the present study. These differences highlight variability in postoperative opioid use and may explain inconsistencies across studies.

The reduction in the median cumulative morphine dose at 24 h with ITM compared with the i.v. analgesia and CTW groups (7 *versus* 14 mg) was modest, but it was accompanied by lower pain scores at rest and during mobilization, indicating enhanced pain control during the critical early postoperative period. This improved pain relief aligns with the goal of minimizing postoperative pain to optimize patient comfort and facilitate early mobilization, even after major surgery. In addition, reducing opioid use may decrease the risk of opioid-induced hyperalgesia. In the present study, ITM was particularly effective during the first 12 h after surgery, significantly reducing pain at rest and during mobilization. The duration of analgesia observed in this study aligns with previous findings^11^. However, pain was not assessed between 12 and 24 h, limiting precise determination of its effect duration. The benefits of intrathecal analgesia could potentially be enhanced by adding a local anaesthetic^12^, which may improve perioperative pain control and reduce the duration of ileus without increasing morbidity. Following the initial ITM effect, an unexpected rebound pain phenomenon was observed. Existing strategies, like those used for rebound pain after locoregional anaesthesia^13,14^, could help reduce this effect but were not applied in the present study.

The ITM analgesic effect is consistent with the findings of Dichtwald *et al.*^15^, who also observed significant early pain reduction with ITM in abdominal surgery. The study by Niewinski *et al*.^16^ also showed similar results, with improved pain control in the first few hours after surgery, but this improvement dissipated after 12 h. The i.v. multimodal strategy used by Niewinski *et al*.^16^, which included systematic postoperative paracetamol and NSAIDs, differs from the present study, which involved perioperative paracetamol and nefopam, followed by systematic paracetamol and rescue nefopam or tramadol. Both the paracetamol–NSAID and paracetamol–nefopam combinations have demonstrated efficacy in pain relief^17^. In the present study, NSAIDs were not used systematically due to concerns about safety regarding acute kidney injury (AKI). AKI is a frequent complication after open liver resection, particularly in patients undergoing major liver resections, as seen for most patients in the present study. The systematic use of NSAIDs after open liver resection, as recommended in the PROSPECT guidelines, seems more suitable for minor liver resections, as evidenced by the references in these guidelines showing rates of 18%^18^, 0%^19^, and unspecified percentages^20^ for major liver resections. No studies have demonstrated the safety of systematic NSAID use after major liver resection, despite a risk of AKI of 20%^21^ in this population, which remains a significant risk for open liver surgery. For patients at high risk of postoperative AKI, ITM combined with paracetamol and nefopam may be an effective early analgesic strategy^2^.

Certain limitations should be considered in this study. No differences in side effects were observed, but this study was not powered to detect potential variations induced by the analgesic techniques used. The lack of intraoperative and postoperative blinding was a potential source of bias. To mitigate this risk, patients were informed neutrally to minimize psychological or performance bias. The cumulative dose of morphine was chosen as the primary outcome because of its objective and quantifiable nature. Pain scores were recorded at relatively wide intervals, potentially missing important trends in the early postoperative period when the effect of ITM is most pronounced. More frequent assessments could have offered a clearer understanding of its duration and the timing of rebound pain. Another limitation is the inclusion period, which extended over 5 years due to the increasing adoption of laparoscopic liver resection. Open laparotomy liver surgery remains the preferred approach for major liver resections, which explains the high rate of over 50% in each group. Antihyperalgesic drugs were not used, as is sometimes the case in major digestive tract surgeries, due to the limited or insufficient evidence supporting their effectiveness and safety specifically in open liver resection at the time of writing and setting up the study^1^. Future studies could explore the potential benefits of adding continuous i.v. lidocaine and antihyperalgesics, such as ketamine, in combination with ITM to improve rehabilitation and comfort after open major liver resection.

In conclusion, the findings of this study highlight the superiority of ITM for the management of early postoperative pain after laparoscopic liver surgery *versus* i.v. analgesia or CWI. With lower pain levels and a morphine-sparing effect compared with CWI and i.v. analgesia in the immediate postoperative period, intrathecal analgesia appears to be a viable alternative for managing pain in patients who have undergone open liver resections. It could be particularly considered for major liver resections when the risk of AKI is significant, and it could serve as a bridge to NSAID use after kidney function has been assessed.

## Supplementary Material

zraf074_Supplementary_Data

## Data Availability

The data that support the findings of this study are available from the corresponding author upon reasonable request.
