# Supplementary material for: Comparison of catheter wound infusion, intrathecal morphine, and intravenous analgesia for postoperative pain management in open liver resection: randomized clinical trial
Source: BJS Open. 2025 Jul 15;9(4):zraf074. doi: 10.1093/bjsopen/zraf074 (PMC12261295; doi:10.1093/bjsopen/zraf074)
Supplement: zraf074_Supplementary_Data [file zraf074_supplementary_data.zip › Study_protocol_Cathepat.pdf]

POST-HEPATECTOMY PAIN MANAGEMENT: CONTINUOUS INFILTRATION OF LOCAL ANESTHETICS VERSUS SPINAL ANALGESIA.

CATHEPAT

Principal investigator :

Pr Gilles Lebuffe, PU-PH Hôpital Claude HURIEZ - Pôle d'anesthésie réanimation Tel: 03 20 44 41 12 Fax: 03 20 44 63 65 Mail : gilles. lebuffe@chru-lille.fr

Promoter:

CHRU de Lille Délégation à la Recherche Clinique et à l'Innovation 2 avenue Oscar Lambret 59037 LILLE Cedex tél : 03 20 44 59 69

Code number assigned by the promoter 2013_48

N° EudraCT 2014-003317-28

Date : 27.10.2014

I. Scientific Justification and General Description of the Research

Postoperative pain (POP) results from an inflammatory process associated with nerve damage secondary to tissue injury. POP manifests as hyperalgesia at the site of injury, defined as primary hyperalgesia, and as secondary hyperalgesia in the periphery of the injury. Some physiological processes that constitute an appropriate response to tissue aggression are the source of neuronal plasticity, which could lead to the emergence of persistent postoperative pain, defined as chronic when it lasts more than two months (1). Postoperative analgesia often involves the use of opioid agents, which are recognized as causing hyperalgesia through the activation of NMDA receptors (2, 3). Indeed, the administration of opioids at doses exceeding the patient's needs gives rise to a "paradoxical" phenomenon known as opioid-induced hyperalgesia. Thus, the use of opioids for pain management is a double-edged sword. They initially provide genuine analgesic effects but simultaneously induce the expression of hyperalgesia through the activation of NMDA receptors, resulting in increased sensitivity to a painful stimulus. Opioids are also responsible for adverse effects that slow the rehabilitation of the operated patient.

Multimodal analgesia, which combines intravenous analgesics with different mechanisms and sites of action, along with regional anesthesia, allows for the reduction of the respective doses and side effects of the analgesics used, notably with a strategy aimed at opioid-sparing (2). The use of continuous infiltration of local anesthetics, regional anesthesia with epidural analgesia, and spinal anesthesia seems to fit into this type of multimodal approach. The broad management of postoperative pain in hepatic surgery is challenging, not only due to the surgical approach but also because of postoperative hepatocellular insufficiency (4). The potential risk of coagulopathy and the benefits of epidural analgesia after major intra-abdominal surgery (gastric, pancreatic, colonic, small intestine) and thoracic surgery for postoperative analgesia are well-documented in the literature (5). However, the specific nature of hepatectomies, with the risk of postoperative coagulopathy, raises concerns about the appearance of an epidural hematoma following epidural analgesia (4).

Patients undergoing hepatic resection often have normal preoperative hemostasis. In patients with cirrhosis, coagulation, even if normal preoperatively, can be quickly disturbed, particularly if the resection is extensive or in the case of "prolonged" vascular clamping. Indeed, hepatic ischemia induced by clamping, and even more so by the state of anhepatic created by the vascular exclusion of the liver, rapidly leads to an alteration (or even cessation) of the synthesis of coagulation factors, resulting in spontaneous hypocoagulation (6). The benefit/risk balance of epidural analgesia in hepatic surgery is therefore debatable (7). Other techniques, such as spinal anesthesia and continuous infiltration of local anesthetics, then become alternatives to epidural analgesia (8, 9, 10).

Spinal anesthesia, performed in the context of hepatic resection with a single dose of intrathecal morphine followed by patient-controlled analgesia (PCA) with morphine, can provide effective and satisfactory postoperative analgesia (11, 12, 13). The quality of this treatment, according to the visual analog scale, is not inferior to continuous epidural analgesia for up to 48 hours after surgery (14). Spinal anesthesia allows for a reduction in perioperative physiological disturbances, such as intraoperative fluid management related to hypovolemia induced by epidural analgesia, and an earlier discharge after hepatic resection (15).

In addition to providing good analgesia, continuous infiltration of local anesthetics is a simple, safe, and cost-effective technique. A systematic review of the literature has shown that

continuous infiltration of local anesthetics is a safe and effective technique in terms of pain score reduction, allowing for opioid-sparing in most surgeries (16, 17). However, the benefit of this technique remains controversial in major abdominal surgery, and this controversy may be related to the position of the catheter. Catheters placed above the fascia provide ineffective analgesia (18), while catheters placed in the preperitoneal space have shown a reduction in postoperative pain and earlier rehabilitation after colorectal surgery (19, 20).

Hepatic surgery is performed via a subcostal approach; the abdominal muscles and the rectus abdominis muscle are firmly adherent to the peritoneum, making it very difficult to access the preperitoneal space in this area. A study conducted in 2010 by S.K. CHAN et al., using a catheter placed inside the musculo-aponeurotic plane of the abdominal wall, demonstrated the analgesic effectiveness of continuous ROPIVACAINE infiltration in hepatic surgery, with a reduction in postoperative opioid consumption, decreased pain at rest and after spirometry, and a less significant reduction in forced vital capacity postoperatively (21).

In the literature, the effectiveness of continuous infiltration of local anesthetics compared to PCA morphine alone is well known. Currently, the two reference techniques for post-hepatectomy analgesia are PCA morphine and spinal analgesia. Epidural analgesia is discouraged due to the risk of postoperative coagulopathy. Therefore, we aim to compare continuous infiltration of local anesthetics with these two reference techniques. We propose to compare continuous infiltration of local anesthetics and spinal analgesia in terms of reducing postoperative morphine consumption and the incidence of chronic postoperative pain at 3 and 6 months after hepatic surgery, compared to the "PCA morphine alone" control group.

The catheter placed inside the musculo-aponeurotic plane of the abdominal wall (in the retromuscular space of the abdominal wall, anterior to the peritoneal plane, more precisely at the anterior surface of the posterior aponeurosis of the rectus abdominis and anterior to the transverse muscle) will therefore be located above the fascia, close to the rectus abdominis muscle, and thus near the preperitoneal space. Finally, the evaluation of postoperative pain intensity (1), the cutaneous surface of peri-scar hyperalgesia (22), and the pain perception threshold using the Pain Matcher® (23) will confirm or refute the predictive nature of these criteria in the occurrence of chronic pain.

II. Objective of the Research

1) Primary Objective

To demonstrate a reduction of at least 30% in postoperative morphine consumption at 24 hours in the "continuous infiltration of local anesthetics + PCA" and "spinal anesthesia + PCA" groups compared to the control group "PCA morphine alone" in hepatic surgery.

2) Secondary Objectives and Generated Hypotheses

- To evaluate postoperative morphine consumption at 48 hours in the "continuous infiltration of local anesthetics + PCA" and "spinal anesthesia + PCA" groups in hepatic surgery.

- To compare the incidence of chronic pain at 3 and 6 months between the "continuous infiltration of local anesthetics + PCA," "spinal anesthesia + PCA," and "PCA morphine alone" groups in hepatic surgery.

- To compare the intensity of pain at rest and during effort at 4, 12, 24, 48, and 72 hours postoperatively between the "continuous infiltration of local anesthetics + PCA," "spinal anesthesia + PCA," and "PCA morphine alone" groups.
- To compare the incidence of postoperative nausea and vomiting between the "continuous infiltration of local anesthetics + PCA," "spinal anesthesia + PCA," and "PCA morphine alone" groups.
- To evaluate the incidence of surgical complications: wound infection, wall abscess, evisceration, bile leakage, hepatocellular insufficiency, ileus, pneumonia, pleurisy, and others in the "continuous infiltration of local anesthetics + PCA" and "spinal anesthesia + PCA" groups

III. Research Design

1) Experimental Plan

Category of the research: Phase III controlled, randomized, open-label efficacy trial, with 3 parallel groups.
Randomization and blinding procedure: Subjects will be randomized according to a randomization table, based on the order of inclusion in the study. The subjects will then receive an inclusion number.
Definition of the studied population

2) Inclusion Criteria

- Male or female over 18 years of age.
- Surgery: hepatic resection surgery via subcostal approach.
- Patient classified as ASA 1 to 3.
- Informed and signed free consent.
- Social security coverage.

3) Non-inclusion Criteria

- Patients classified as ASA 4 or 5.
- Allergy or intolerance to one of the products used during the protocol.
- Emergency surgery, palliative surgery, or reoperation.
- History of chronic pain requiring regular analgesic use, especially opioids.
- History of medication misuse.
- Inability to understand and/or use patient-controlled analgesia (PCA) morphine pump.
- Known history of psychiatric disorders or current psychotropic treatment (excluding benzodiazepine monotherapy).
- Addiction to alcohol or psychodysleptic substances that has not been treated.
- Contraindication to morphine treatment.
- Contraindication to an anesthetic protocol treatment.
- Contraindication to a treatment used during the study.
- Minor or legally incapacitated adult.
- Intellectual incapacity preventing a good understanding of the protocol.
- Uncooperative patient or refusal to sign the informed consent letter.
- Pregnant or breastfeeding woman.
- Lack of social security coverage.

4) Evaluation Criteria

Primary criterion:

- Morphine consumption during the first 24 hours.

Secondary criterion (criteria):

- Morphine consumption during the first 48 postoperative hours.
- Incidence of chronic pain at 3 and 6 months in hepatic surgery.
- Postoperative acute pain intensity on the numeric scale (NS) at rest and during effort; pain perception threshold with Pain Matcher®.
- Surface area of the peri-scar hyperalgesia zone.
- Incidence of postoperative nausea and vomiting.
- Incidence of adverse effects related to postoperative morphine use.
- Time to return of intestinal transit and resumption of normal diet.
- Duration of hospital stay.

Evaluation of efficacy:
Preoperatively and postoperatively, pain intensity will be assessed using several methods: Numeric scale, Pain Matcher® (electric stimulation device), and questionnaires.

Evaluation of safety:
Throughout the postoperative period, adverse effects and potential complications from the treatments used will be recorded and reported (see corresponding chapter). Continuous clinical monitoring, as well as blood pressure and pulse oximetry monitoring, will be systematically performed during the first 48 postoperative hours.

5) Sample Size Calculation

The calculation of the required number of subjects was based on the expected difference in the primary endpoint, which is total morphine consumption at 24 hours. The aim of the study is to compare two "Treated" groups to a third group, the "Control" group. Therefore, two statistical tests will be performed. In the S.K. Chan study on the effect of continuous Ropivacaine infiltration via a multi-perforated catheter in hepatic surgery (21), the total morphine consumption at 24 hours was 40 +/- 20 mg. We hope to demonstrate a 30% reduction in this consumption between each treated group and the control group, i.e., 12 mg. Considering an α-risk of 0.025 (α-risk correction), an expected difference of 12, a standard deviation of 20, and a power of 80%, under the assumption of a two-sided Student test, exactly 55 subjects per group must be included.

6) Method and Analysis Strategy

The following statistical analyses will be performed:

1. **Descriptive analysis:** Numerical variables will be described by mean and standard deviation in the case of a Gaussian distribution and by mean, median, and quartiles otherwise. The normality test will be performed using the Shapiro-Wilk test. Qualitative variables will be described by frequencies and percentages.

2. **Analysis of the primary objective:** The primary endpoint will be analyzed using a one-factor ANOVA. Differences between each treated group and the control group will be

tested using contrasts with Dunnett's correction. The difference between the two treated groups will be provided with its 95% confidence interval.

3. **Comparison of frequencies:** Frequency comparisons will be made with the Chi-square test, with or without Fisher's exact test if the Chi-square is not applicable.
4. **Morphine consumption measured at several time points (postoperative, Day 1, Day 2, and Day 3):** The time course of morphine consumption in the three groups will be analyzed using a mixed model, with the use of contrasts. The same statistical methods will be used for the analysis of secondary endpoints.

The statistical analysis will be carried out by the Methodological Support Platform (Prof. Duhamel) using SAS software.

IV. Study Logistics

1) Participating Teams and Team's Experience in the Field

The investigator anesthesiologists mentioned on the first page, from the anesthesia and intensive care unit of Claude Hurlez hospital. The medical staff from the Surgical Unit and the central operating room of Claude Huriez hospital, CHRU Lille (nurses, nursing assistants, physiotherapists, psychologists).

2) Study Location

The study will take place in the Surgical Unit and the central operating room of Claude Huriez hospital, CHRU Lille. The evaluation of chronic pain will be carried out using questionnaires (QDSA, DN2, QEDN) (appendix 4, appendix 5, appendix 6), sent by mail to the patients' homes at 3 and 6 months. Patients may be referred to the Pain Consultation of the Anesthesia and Intensive Care Unit of Claude Huriez hospital in case of persistent, debilitating pain after discharge from the hospital.

3) Practical Conduct of the Study

Patient Reception = Visit No. 1 (between Day -30 and Day -3

The consultation will take place in the anesthesia and intensive care clinic at Huriez within three days to one month before surgery. During the anesthesia consultation and after verifying eligibility criteria, the study will be presented to the patient:

- The use of the numeric pain evaluation scale (NS) will be explained.
- The functioning of the Pain Matcher® will be explained. The Pain Matcher® is a device that estimates, quantifies, and records pain, determining a somatosensory and pain threshold. The usefulness of this measure has been validated in surgery (23, 24). The Pain Matcher® pain perception threshold (23) is predictive of the incidence of chronic pain. The ability to use the device will be checked, and two measurements will be performed to ensure the patient is familiar with the device and the procedure.
- The patient will be informed about the peri-scar hyperalgesia measurement technique using a 10g Von Frey hair. The surface area of peri-scar hyperalgesia has been described

as predictive of postoperative chronic pain. The chronic pain questionnaires (QDSA, QEDN, DN2) will be presented and explained. The patient-controlled morphine analgesia (PCA) system will be explained, and the patient's ability to use this equipment will be verified. The information letter and informed consent form will be given to the patient (appendix 1, appendix 2).

Pre-anesthesia Visit and Inclusion = Visit No. 2 (Day -1)

In the adult digestive and transplant surgery hospitalization units at Huriez, the day before the surgery. During the pre-anesthesia visit, eligible patients will be included in the study (signature of consent after a reflection period between the consultation and the pre-anesthesia visit). The study's principles will be explained again to the patient, who will return the signed informed consent letter. At this time, a pain intensity measurement (NS) and a Pain Matcher® pain threshold measurement will be performed. Pain intensity (NS) will be measured first to evaluate after applying an unpleasant electrical stimulation. The patient will be asked to complete the different questionnaires used in this study (QDSA, QEDN, DN2) to recognize preoperative pain and compare responses at 3 and 6 months. Results will be recorded in the observation notebook. Randomization will be performed by the investigator doctor in the Anesthesia and Intensive Care Unit of Claude Huriez hospital or by one of the study's co-investigators. Patients will be allocated to each group based on a randomization table balanced in blocks of 6. The investigator will follow the order of the randomization envelopes and assign the patient to the corresponding group.

Group I: Control Group
Group II: Continuous Infiltration of Local Anesthetics
Group III: Spinal Analgesia

Day before the surgery

All patients included in the protocol will be hospitalized the day before the surgery. They will fast starting at midnight, and premedication with sublingual Midazolam 0.05 to 0.1 mg/kg will be given the night before and on the morning of the surgery, no later than one hour before the procedure.

Operative Period = Visit No. 3 (Day of Surgery, Operating Room)

All patients will receive a standardized anesthesia protocol with entropy measurement to ensure equal anesthesia depth between the groups.
Induction: After pre-oxygenation for a Fet O2 > 90%, Propofol will be administered intravenously (IV) at a dose of 2 to 4 mg/kg, combined with sufentanil at a dose of 0.2 to 0.3 µg/kg and atracuriumat a dose of 0.5 mg/kg.

Maintenance: Continuous IV Propofol via Diprifusor® will be used to maintain entropy between 40 and 60. Halogenated volatile anesthetics will be prohibited due to their probable action on NMDA receptors and postoperative hyperalgesia. Sufentanil may be administered in IV boluses of 5 µg to 10 µg as per routine clinical practice (for a >20% variation from baseline systolic blood pressure and heart rate). The last injection should be given no later than 30 minutes before the end of the surgery. Atracurium reinjections will be guided by curarization monitoring at one-third of the induction dose. Patients will be ventilated with an oxygen/air mixture for a Fet CO2 between 30 and 50 mm Hg, in a closed circuit.

Analgesia: Thirty minutes before the end of the surgery, 1 gram of paracetamol and 20 mg of Acupan will be administered intravenously. At the end of the surgery, patients will be awakened

and extubated in the operating room, provided there is no residual curarization and they are normothermic, before being transferred to the postoperative recovery unit. The total intraoperative sufentanil consumption will be recorded.

In the **control group (Group I),** patients will not receive spinal analgesia or continuous infiltration of local anesthetics.
In the **spinal analgesia group (Group III),** patients will receive an intrathecal injection of 300 µg of morphine preoperatively, just before anesthesia induction.

Procedure:

Before anesthesia induction and after careful back disinfection, the patient will be placed in a seated position with a well-flexed back. After wearing a mask, cap, performing surgical hand washing, and donning sterile gloves, the doctor will perform a puncture at the L3-L4 or L4-L5 level using a spinal puncture needle with an introducer ("Whitacre type ref. 181.06, diameter 0.53 mm, 25G and L. S0mm"), after possible local anesthesia. Once cerebrospinal fluid return is visualized in the needle, 300 µg of morphine (diluted 1/100 in 0.9% saline) will be injected, and the equipment will be removed.

In the **continuous infiltration of local anesthetics group (Group II),** before the intermediate surgical closure, a multi-perforated catheter with a connector and filter ("Profilis Paincath P500-30QR" catheter) will be placed in the retromuscular space of the abdominal wall, anterior to the peritoneal plane, specifically at the anterior surface of the posterior aponeurosis of the rectus abdominis and anterior to the transverse muscle. This will be performed by the surgeon using a 4F peelable introducer in a sterile manner. The catheter is perforated with micro-holes at 2.5, 5, 7.5, 10, 15, 20, 25, or 30 cm intervals, with the distal end closed and the other end open for connection to a pre-mounted connector. The device comes in sterile packaging. A bolus of 20 ml of 2% Ropivacaine (2 mg/ml) will be administered, followed by a maintenance dose of 8 ml per hour.

Postoperative Period = Visit No. 4 (Day of Surgery, Recovery Room)

Upon arrival in the recovery room, systematic evaluations will include: pain intensity assessment using a numeric score from 0 to 10 (NS) and initiation of PCA morphine following the usual protocol of the department (syringe preparation: 50 mg of morphine in 50 ml, i.e., 1 mg/ml + droleptan 2.5 mg in 50 ml, i.e., 0.05 mg/ml; bolus of 1 ml, refractory period of 5 minutes, no maximum dose). The start time of the PCA will be recorded. Upon leaving the recovery room, the patient will be hospitalized in either the postoperative intensive care unit (SIPO) or continuous care unit, depending on the surgery and the patient's medical history. Continuous clinical monitoring, as well as blood pressure and pulse oximetry monitoring, will be performed during the first 48 postoperative hours. The patient will receive analgesia with 1g of paracetamol four times a day, and 20 mg of Acupan up to six times a day if needed for NS > 3/10, and 50 mg of tramadol up to four times a day if needed for NS > 4/10.

Postoperative Hospitalization Period

The following will be measured:

- Morphine consumption by PCA.
- Pain at rest, during coughing, and mobilization (tilting the bed headrest to 45°). These will be measured after recovery room discharge, every 3 hours for 48 hours, and at 72 hours.

- The pain perception threshold using the Pain Matcher® will be measured during the pre-anesthesia visit, then upon leaving the recovery room, at 24h, and at 48h.
- Adverse effects related to morphine use (nausea, vomiting, pruritus, drowsiness, confusion, etc.) will also be recorded, along with the time and date of the first gas and stool passage.
- Surgeons will prescribe the time for nasogastric tube removal, the date of the first "normal" meal (D3 according to the surgical protocol), and the discharge date.

On the second day, the peri-scar hyperalgesia skin surface area (in cm^2) will be measured using a 10g Von Frey filament, a technique previously validated.
We will also perform comparative, quantitative, and qualitative measurements of postoperative pain using three specific and validated questionnaires: QDSA, QEDN, DN2 (25, 26, 27).

Patient Follow-up

At three and six months, we will evaluate the incidence of chronic pain. Patients will be contacted by phone by the principal investigator and/or a co-investigator, and will receive the previously mentioned questionnaires (along with a pre-paid envelope addressed to the department) to complete and return. These questionnaires will be anonymized, but patient inclusion numbers will allow data collection and patient identification. The investigation results will be recorded in the observation notebook. In the event of no response from a patient, follow-up procedures will include reminders, calls to the patient's home, and calls to their general practitioner.

4) Duration

The inclusion period will last two years, with a follow-up period of six months. The total study duration will be three years.

The sponsor reserves the right to stop the trial due to a lack of inclusion. The study may be discontinued by joint decision of the authorities.

5) Criteria for Discontinuing Participation in the Study

Early termination criteria: The subject may stop participating in the study at any time without justification. In addition, the investigator must withdraw from the study:

- Any subject not adhering to the protocol.
- Subjects experiencing adverse events preventing continuation of treatment.
Each subject may be withdrawn from the study by decision of the administrative authority, the sponsor, the coordinating investigator, a co-investigator, or by the subject themselves, in accordance with regulations and as mentioned in the consent form.

6) Prohibition of Simultaneous Participation - Exclusion Period

Simultaneous participation in another drug-related research study for pain management is prohibited 10 days before inclusion and throughout participation in the study. In this case, the patient should be registered in the national database of individuals participating in biomedical research and informed of this registration.

7) Study Benefits, Risks, and Constraints

The expected benefits include reduced postoperative morphine consumption and its adverse effects compared to the control group (Group I), leading to less postoperative pain and earlier return of bowel function, which may reduce the length of hospitalization. Continuous infiltration of local anesthetics avoids the complications of perimedullary anesthesia while providing effective postoperative analgesia. The foreseeable risks of the protocol include the usual surgical or anesthetic complications of hepatic resection via subcostal incision, such as thrombophlebitis, pulmonary embolism, wound infection, or pneumonia. The foreseeable risks of continuous Ropivacaine infiltration include the occurrence of bradycardia or hypotension, or even cardiac arrest, in case of intravascular passage, monitored via blood pressure monitoring. The investigator will be immediately notified and decide whether to stop Ropivacaine administration and institute treatment. Neurological complications such as seizures may also occur in case of intravascular passage and will be treated according to recommendations. Such adverse events will result in the immediate discontinuation of Ropivacaine infusion and the patient's withdrawal from the protocol. There is also an infectious risk due to the continuous presence of a catheter, potentially leading to wall infection and operative site infection by contiguity. The foreseeable risks of spinal analgesia include bradypnea, respiratory distress, acute urinary retention, nausea/vomiting, and pruritus.

8) Independent Monitoring Committee

PCA morphine and spinal analgesia are commonly used techniques in hepatic surgery. Continuous infiltration of local anesthetics by catheter is frequently used in major abdominal surgery, urologic surgery, and certain orthopedic surgeries, and no significant adverse effects are expected with this technique.

Given the current knowledge of the benefit/risk ratio of the study, there is no plan to establish a monitoring committee.

V. V. Treatment Administered

1) Medications Used

ROPIVACAINE KABI 2 mg/ml,

Injectable solution, Ropivacaine hydrochloride, 40 mg/20 ml vial. Composition: 1 ml of solution contains 2.12 mg of ropivacaine hydrochloride monohydrate (corresponding to 2 mg of ropivacaine hydrochloride).

Indications:
Ropivacaine hydrochloride is a local anesthetic solution intended for parietal infiltration anesthesia, epidural analgesia, regional anesthesia, and nerve block anesthesia.
Contraindications:

- Known hypersensitivity to ropivacaine hydrochloride, amide-linked local anesthetics, or any of the excipients.
- Intravenous regional anesthesia.
- Paracervical obstetric anesthesia.

- Hypovolemia.

Dosage and Administration:

Ropivacaine 2 mg/ml will be administered using a multi-perforated catheter with connector and filter ("Cathéter Profilis Paincath P500-30QR") before intermediate surgical closure in a sterile environment by the surgeon. A bolus of 20 ml of Ropivacaine will be administered, followed by a continuous infusion at a rate of 8 ml per hour. This infusion will last for the duration of the surgery and up to 48 hours after its completion.

Expected Side Effects:

Cardiovascular (hypotension, bradycardia), central nervous system (paresthesia or peripheral numbness, dysarthria, tremors, hypoesthesia).

MORPHINE CHLORHYDRATE LAVOISIER,

Injectable IV solution, 10 mg/mL ampoule (1 mL = 10 mg).

Indications:

Severe and/or refractory pain that requires continuous morphine administration using programmable medical devices.

Contraindications:

- Hypersensitivity to morphine.
- Decompensated respiratory insufficiency.
- Severe hepatocellular insufficiency.
- Uncontrolled epilepsy.
- Concomitant use with buprenorphine, nalbuphine, or pentazocine.
- Breastfeeding in cases of long-term treatment initiation or continuation after birth.

Dosage and Administration:

Morphine will be administered via PCA (patient-controlled analgesia) at a concentration of 1 mg/ml. Boluses will be 1 ml, with a refractory period of 5 minutes, without a maximum dose or continuous background infusion. This will be maintained for the first 48 postoperative hours.

Expected Side Effects: Drowsiness, confusion, nausea, vomiting, constipation, respiratory depression, increased intracranial pressure, dysuria, urinary retention, pruritus, flushing, and withdrawal syndrome.

Spinal Analgesia (Rachianalgesia):

Intrathecal injection of 300 micrograms of morphine preoperatively, just before anesthesia induction.

Expected Side Effects: Bradypnea, respiratory distress, acute urinary retention, nausea/vomiting, pruritus, compressive hematoma, infectious complications, and headaches.

DROLEPTAN® Injectable Solution 2.5 mg/ml, Droperidol

Injectable IV solution, 2.5 mg/ml: Ampoules of 1 ml, box of 10.

Indications:

Treatment of postoperative nausea and vomiting in adults and children. Prevention of morphine-induced nausea and vomiting in postoperative patient-controlled analgesia in adults.

Contraindications:

- Known hypersensitivity to droperidol or any component of the product.
- Comatose states.
- Known hypokalemia.

- Bradycardia below 55 beats per minute.
- Knowledge of ongoing treatment with medications causing bradycardia, slowed intracardiac conduction, or prolonged QT interval.
- Severe depressive syndrome.
- Pheochromocytoma.
- Breastfeeding.
- Sultopride.

Dosage and Administration:
Postoperative nausea and vomiting treatment in adults: 0.625 mg to 1.25 mg IV, titrated. Lower doses are generally effective, with a maximum dose of 2.5 mg if needed. For prevention of morphine-induced nausea and vomiting in postoperative patient-controlled analgesia, each 1 mg morphine bolus will be paired with a 0.05 to 0.1 mg droperidol IV bolus. If necessary, this may be preceded by a single IV bolus of 0.625 to 1.25 mg, up to a maximum of 2.5 mg.
Expected Side Effects: Indifference, anxiety reactions, mood changes (neuropsychic disorders), reflex tachycardia (cardiovascular disorders), impotence, frigidity (endocrine and metabolic disorders), orthostatic hypotension (neuro-vegetative disorders), rash, exanthem, or anaphylactic reactions, and in rare cases, inappropriate antidiuretic hormone secretion and angioedema, particularly of the tongue.

2) Treatment Scheme and Duration

In the control group (Group I), patients will receive patient-controlled analgesia (PCA) with morphine only in the postoperative period. In Group II, patients will receive continuous infiltration of Ropivacaine 2 mg/ml at a rate of 8 ml per hour through a multi-perforated catheter with a connector and filter ("Cathéter Profilis Paincath P500-30QR"), placed before intermediate surgical closure by the surgeon. This catheter will be removed 48 hours postoperatively. They will also receive PCA morphine postoperatively.
In the **spinal analgesia group (Group III),** patients will receive a single intrathecal injection of 300 micrograms of morphine preoperatively, just before anesthesia induction. They will also receive PCA morphine postoperatively.

3) Presentation of the Medication Used

A 50 ml syringe containing 2% Ropivacaine (2 mg/ml) will be connected to the multi-perforated catheter with a filter ("Cathéter Profilis Paincath P500-30QR"). The solution will be administered using an auto-pulsed syringe, directly connected to the catheter.

4) Medication Accountability Procedures and Compliance Monitoring Method

Ropivacaine and Droperidol will be dispensed as partial supplies, renewed based on inclusions. Renewals will be made through a request signed by an investigator. Morphine will be dispensed as partial supplies, transported in a narcotics case dedicated to the study. The renewal of morphine ampoules will be done through prescription using a stub book with administration traceability, signed by a study investigator, as well as the primary packaging (empty ampoules). Morphine will be transported to the department in a narcotics case. Once in the department, morphine will be stored in the narcotics safe in a separate compartment from other ampoules. The medications used will be recorded in each patient's observation

notebook. Product traceability will be ensured by recording lot numbers. The experimental medications will be labeled in accordance with current regulations.

VI. VI. Adverse Events

1) Definitions

- **Adverse Event:** Any harmful occurrence in a person participating in biomedical research, whether or not related to the research or the product being studied.
- **Adverse Effect:** Any adverse event due to the research.
- **Serious Adverse Event or Effect:** Any adverse event or effect that:
  - Results in death,
  - Puts the participant's life at risk,
  - Requires hospitalization or prolongation of an existing hospitalization,
  - Causes significant or lasting disability or incapacity, or results in congenital anomaly or malformation,
  - Is considered medically serious by the investigator.
- **Unexpected Adverse Effect:** Any adverse effect whose nature, severity, or course does not match the information about the products, procedures, or methods used in the research.

2) Adverse Events and Risks Related to the Protocol

The foreseeable risks related to the protocol are represented by the usual surgical or anesthetic complications of a hepatic resection via subcostal approach, such as thrombophlebitis, pulmonary embolism, wound infection, or pneumonia. The side effects of Ropivacaine will be monitored. The occurrence of cardiac rhythm disturbances such as bradycardia or hypotension, or even cardiovascular arrest, in case of intravascular passage, will be monitored by blood pressure monitoring. The investigator will be immediately notified and will decide whether to stop the Ropivacaine infusion and administer treatment. The occurrence of neurological issues, such as seizures in the case of intravascular passage, will also be monitored and treated by the investigator according to the recommendations for seizure treatment. This type of adverse effect will result in the immediate discontinuation of the Ropivacaine infusion and the patient’s removal from the protocol. The infectious risk associated with the continuous presence of a catheter, which could cause wound or operative site infections by contiguity, will also be monitored. All serious adverse events will be recorded in the observation notebook (Appendix 7).

3) Procedures for Recording and Reporting Adverse Events Investigator Responsibilities:

The investigator must notify the sponsor immediately upon becoming aware of any serious adverse events during the trial. All serious adverse events must be reported using the “Serious Adverse Event” form found in the observation notebook. This form must be sent to the sponsor (Vigilance Cell of the Clinical Research Federation) by fax at 03 20 44 57 11. For each adverse event, the investigator assesses its severity and the causal relationship between the adverse event and the product(s) or protocol being studied. The investigator will ensure follow-up for adverse events.

Sponsor Responsibilities:

- **Reporting Serious Adverse Events and Effects:**

For each serious adverse event or effect, the sponsor assesses its severity, the causal link to the product(s) being studied or the protocol, and whether it is unexpected. The sponsor is responsible for reporting any serious, unexpected adverse events related to the study product(s) or procedures to the ANSM (French National Agency for Medicines and Health Products Safety) and the CPP (Committee for the Protection of Persons) within 15 days (7 days in the event of death or life-threatening situations).

- **Notification to Investigators:**

The sponsor will inform all study investigators of any serious adverse events or effects that may negatively impact participant safety.

- **Annual Safety Report:**

Once a year throughout the trial or upon request, the sponsor will submit a safety report to the ANSM and the CPP. This report will include a global safety analysis of the study protocol, taking into account any new relevant safety data. The safety information will be presented in summary tables outlining the serious adverse events or effects that have occurred in the biomedical research.

VII. Right of Access to Data and Source Documents

The investigator agrees to allow monitoring by the sponsor (monitor and/or auditor) or inspection by the competent administrative authority. They ensure access to source data (medical records, computer files, study documents, etc.).

VIII. Monitoring of the Study

The medical observations will be stored in the patient's file, and clinical data detailing the various stages of the study will be recorded in the study's observation notebooks, following best practices. Any deviation from the protocol will be documented, along with the reason for the patient's management within the protocol. Data collection will be comprehensive and regularly checked by a Clinical Research Assistant following the protocol's procedures.
The study monitoring will be conducted according to the monitoring plan validated before the start of the research or triggered by a specific request from the sponsor's Clinical Research Assistant (CRA). A setup meeting with the principal investigator will be held before the start of the trial (recall of GCP [Good Clinical Practice], research organization, planned monitoring). The investigator will inform the sponsor in real-time of inclusions.
During monitoring visits to the site, CRAs must be able to review the patient data collection notebooks, medical and nursing records, and the investigator's folder.
The monitoring will at least check the following: patient existence, consent forms, adherence to inclusion criteria, primary judgment criteria, monitoring and reporting of serious adverse events, new developments requiring an amendment, and pharmacy monitoring.

Closing the Study

At the end of the trial, closing procedures will be applied, with all documents and source data filed. Once the final analysis has been completed and validated, all the documents and data will be sealed and archived in secure premises according to specific procedures.

IX. Ethical and Legal Considerations

The research will be conducted according to the protocol, Good Clinical Practice, and applicable legal and regulatory provisions. The investigator is responsible for conducting the trial. The investigator agrees to:

- Keep source data and administrative documents related to the protocol,
- Not include volunteers before receiving official authorizations from the CPP and the competent authority,
- Follow the protocol,
- Conduct the study according to moral, regulatory, ethical, and scientific principles governing clinical research,
- Obtain written informed consent from each volunteer,
- Report any serious adverse event.

Participants will receive complete oral and written information about the course of the trial. An information letter will be given to the participant by the investigator or the physician representing them before their inclusion in the study. A signed informed consent form (annexed to the protocol) will be obtained from each participant before they are included in the study. No protocol-specific actions should begin without the patient's signed consent.
The information letter and consent form will be prepared in three copies: one will be given to the participant, one will be kept by the investigator, and the last will be sent to the sponsor in a sealed envelope (designed so that it cannot be resealed after opening).
The consent form will be signed by both the investigator or the physician representing them and the patient.
Any person agreeing to participate in this study will be registered by the investigator in the national database of people participating in biomedical research. This registration will be carried out in strict compliance with the decree of November 14, 2006, relating to the data constituting the national database of people participating in biomedical research.

Authorization from the Competent Authority and Opinion of the CPP

The sponsor will submit an authorization request to the ANSM and obtain a favorable opinion from the CPP before the start of the research, in accordance with Article L1121-4 of the French Public Health Code.

Protocol Amendments

Only the sponsor, in consultation with the coordinating investigator, is authorized to modify the protocol.
Substantial amendments refer to changes that have a significant impact on any aspect of the research, including participant protection (especially regarding safety), research validity, product quality and safety, interpretation of scientific documents supporting the research, or its conduct. A request for a substantial amendment is sent by the sponsor to either the ANSM, the CPP, or both, depending on the case, for authorization and/or opinion. Upon receipt of the authorization and/or favorable opinion, the amended version of the protocol is sent to all investigators by the sponsor.
Non-substantial amendments are minor changes or clarifications that do not affect the conduct of the trial. These amendments will not be submitted to the competent authorities but will be agreed upon between the sponsor and the investigator and will be clearly documented (in the study follow-up file).

X. Data Processing and Retention of Research-Related Documents and Data

The processing of data will be carried out under the confidentiality conditions defined by the modified law of January 6, 1978, relating to information technology, files, and freedoms (CNIL). Data processing will be conducted in accordance with the requirements of the CNIL's reference methodology MR 06001 (see CNIL Appendix). To maintain data anonymity, the names of subjects entered into the study will only be identified by the first two letters of their last name and the first two letters of their first name by the investigator. The investigator will retain a list for their own use, linking the subjects' identities, addresses, phone numbers, and file numbers to the numbers, initials, or codes under which they appear in the trial documents. This list will be archived along with the trial documents. Data related to this study will be archived for a minimum period of fifteen years from the end of the research or its early termination, without prejudice to the legal and regulatory provisions in force.

XI. Funding and Insurance

1) Funding

The cost of the prescribed medications will be borne by the Anesthesia Clinic of CHRU Lille. Material-related expenses, such as observation notebooks, questionnaire copies, and pre-stamped envelopes for returning the questionnaires, will amount to 3,000 euros for the study. **Funding for a Clinical Research Assistant (CRA):** No.

2) Insurance

The sponsor will take out an insurance policy to cover their civil liability and that of all participants in the study, in accordance with Article L1121-10 of the Public Health Code.

XII. Publication and Exploitation

The final report of the study will be drafted by the sponsor. The principal investigator, Prof. Lebuffe, will send it to the sponsor. In accordance with Article R 5121-13 of the Public Health Code, no written or oral comments on the trial may be made without the joint agreement of the investigator and the sponsor. Any publication must mention that the CHRU Lille is the sponsor (Identification No. 2013-48°, to be requested from the Research Delegation). In any case, CHRU Lille, as the sponsor of the study, retains control over the first publication. The investigator must send a copy of any publications to the sponsor. The sponsor is the exclusive owner of the study results. These results, as well as all research-related data, must not be transmitted to third parties under any circumstances, without a negotiated prior agreement by the Research Delegation. Any such request must be promptly forwarded to the legal department of the Research Delegation.

XIII. References

1/ Frederick M. Perkins, Henrik Kehlet. Chronic Pain as an Outcome of Surgery A Review of Predictive Factors Anesthesiology 2000; 93: 1 123-33

2/Koppert W Opoid-induced hyperalgesia. Pathophysiology and clinical relevance. Anaesthesist. 2004 May; 53(5): 455-6. 3/ Koppert W, Schmelz M. The impact of opoid-induced hyperalgesia for postoperative pain. Best Pract Res Clin Anesthesiol.2007 Mar,; 21(1): 65-83.

4/ Flora Shiyi Yuan, Shin Yi Ng. Kok Yuen HO, Ser Yee Lee Alexander Y. Chung, Ruban Poopalalingam Abnormal coagulation profile after hepatic resection: the effect of chronic hepatic disease and implications for epidural analgesia Journal of Clinical Anesthesia (2012) 24, 398-403

5/D. Fletcher,C. Jayr Indications of epidural analgesia Annales Françaises d'Anesthésie et de Réanimation 28 (2009) e95-e1246

6/ Liver surgery and anesthesiology Le Praticien en anesthésie réanimation (2009) 13, 418-428

7/ Erica J. Revie et al. Effectiveness of epidural analgesia following open liver resection HPB 2011, 13,206-211

8/ S Basu, A Tamijmarane, D Bulters, JKG Wells, TG John and M Rees An alternative method of wound pain control following hepatic resection: a preliminary study HPB 2004 Volume 6, Number 3 186t189 9/ Erica J. Revie, Dermot W. McKeown, John A. Wilson, O. James Garden & Stephen J. Wigmore Randomized clinical trial of local infiltration plus patient-controlled opiate analgesia vs. epidural analgesia following liver resection surgery HPB 2012, 14,611-618

10/ Molly E. Gross, Edward T. Nelson, Mary C. Mone, Heidi J. Hansen, Bradford Sklow, Robert Glasgow, Courtney L. Scaife. A comparison of postoperative outcomes utilizing a continuous preperitoneal infusion versus epidural for midline laparotomy The American Journal of Surgery (2011) 202, 765-770

11/ Justin Sangwook Ko, Soo Joo Choi, Mi Sook Gwak, Gaab Soo Kim, Hyun Joo Ahn, Jie Ae Kim, Tae Soo Hahm, Hyun Sung Cho, Kyoung Mi Kim, and Jae Won Joh Intrathecal Morphine Combined with Intravenous Patient-Controlled Analgesia Is an Effective and Safe Method for Immediate Postoperative Pain Control in Live Liver Donors Liver Transplantation 15:381-389, 2009

12/ Jean-Denis Roy et al. A Comparison of Intrathecal Morphine/Fentany| and Patient-Controlled Analgesia with Patient- Controlled Analgesia Alone for Analgesia After Liver Resection Anesth Analg 2006; 103:990-4

13/ Jean-Michel Devys, Anne Mora, Benoit Plaud, Christian Jayr, Agnès Laplanche, Bruno Raynard, Philippe Lasser, Bertrand Debaene Version n 2 du 27.10.2014 26

14/Intrathecal + PCA morphine improves analgesia during the first 24 hr after major abdominal surgery compared to PCA alone Can J Anesth 2003 / 50: 4/ pp 355-361 14/ Lesley De Pietri, Antonio Siniscalchi, Alexia Reggiani, Michele Masetti, Bruno Begliomini, Matteo Gazzi, Giorgio E. Gerunda, and Alberto Pasetto, The Use of intrathecal Morphine for Postoperative Pain Relief After Liver Resection: A Comparison with Epidural Analgesia Anesth Analg 2006; 102:1157-63

15/ Jonathan B Koea, Yatin Young, and Kerry Gunn Fast Track Liver Resection: The Effect of a Comprehensive Care Package and Analgesia with Single Dose Intrathecal Morphine with Gabapentin or Continuous Epidural Analgesia HBP surgery 2009

16/ Liu SS, Richman JM, Thiriby RC, Wu CL. Efficacy of continuous wound catheters delivering local anesthetic for postoperative analgesia: a quantitative and qualitative systematic review of randomized controlled trials Journal of the american college of surgeons 2006; 203: 914-3

17/ Félix Lluis et al. Safety of a Multiperforated Catheter Implanted in the Surgical Wound for the Continuous Infusion of Local Anaesthetics in Post-Operative Analgesia CIR ESP. 2011;89(9):613-617

18/ Fredman B, Zohar E, Tarabykin y et al. Bupivacaine wound instillation via an electronic patient-controlled analgesia device and a double- catheter system does not decrease postoperative pain or opiod requirements after major abdominal surgery. Anesthesia and Analgesia 2001; 92 : 189-93

19/ Marc Beaussier et al. Continuous Preperitoneal Infusion of Ropivacaine Provides Effective Analgesia and Accelerates Recovery after Colorectal Surgery Anesthesiology 2007; 107:461-8

20/ Sergio Bertoglio et al. The Postoperative Analgesic Efficacy of Preperitoneal Continuous Wound Infusion Compared to Epidural Continuous Infusion with Local Anesthetics after Colorectal Cancer Surgery: A Randomized Controlled Multicenter Study Anesthesia and analgesia December 2012. Volume 115. Number 6

21/S. K. Chan, P. B. Lai, Q T Li, J. Wong, M. K. Karmakar, K. F. Lee and K Gin The analy lgesic efficacy of continuous wound instillation with ropivacaine after open hepatic surgery Anaesthesia, 2010, 65, pages 1180-1186

22/ Marc De Kock, Patricia Lavand'homme, and Hilde Waterloos. The short- Lasting Analgesia and Long-Term Antihyperalgesic Effect of Intrathecal Clonidine in Patient Undergoing Colonic Surgery. Anesth Analg 2005; 101:566-72

23/ Nielsen PR, Nergaard L, Rasmussen LS, Kehlet H Prediction of post-operative pain by an electrical pain stimulus. Acta Anaesthesiol Scand. 2007 May;51(5):582-6

24/ Alstergren P, Forstrom J Acute oral pain intensity and pain threshold assessed by intensity matching to pain induced by electrical stimuli.

25/ Boureau F, Luu M, Doubrère JF. Comparative study of the validity of four French MeGill Pain Questionnaire (MGP) version. Pain.1992 Jul; 50(1): 59-65. Comparison of pain syndrome associated with nervous or stormnatic lesions and development of a new

26/ Bouhassira D, and ai. Comparison of pain syndrome associated with nervous or stomatic lesions and development of a new neuropathic pain diagnositic questionnaire DNA. Pain.2005 Mar; 114(1-2): 29-86.

27/ Bouhassira D, and al. Development and validation of the Neuropathic Pain Symptom Inventory Pain.2004 apr; 100(3): 248-57
