# Supplementary material for: Comparison of catheter wound infusion, intrathecal morphine, and intravenous analgesia for postoperative pain management in open liver resection: randomized clinical trial
Source: BJS Open. 2025 Jul 15;9(4):zraf074. doi: 10.1093/bjsopen/zraf074 (PMC12261295; doi:10.1093/bjsopen/zraf074)
Supplement: zraf074_Supplementary_Data [file zraf074_supplementary_data.zip › Supplementary_Material.docx]

**Title :**

Comparison of Catheter Wound Infusion, Intrathecal Morphine, and Intravenous Analgesic for Postoperative Pain Management in Open Liver Resection: Randomized Controlled Trial.

**Authors :**

Damien ROUSSELEAU, MD, (1,2)

Barthélémy PLANE, MD, (1)

Julien LABREUCHE, Biostatistician (3)

Adeline PIERACHE, Biostatistician (3)

Younes EL AMINE, MD, (1)

Sabine ETHGEN, MD, (1)

Jean-Michel WATTIER, MD (1)

Cédric CIRENEI, MD (1,4)

Emmanuel BOLESLAWSKI, MD/PhD, (5)

Gilles LEBUFFE, MD/PhD (1, 2)

**Department :**

(1) Department of Anesthesiology and Critical Care, Hospital Claude Huriez, Lille University Hospital, F59000-Lille, France

(2) ULR 7365-GRITA-Groupe de Recherche sur les formes Injectables et les Technologies Associées, Lille University, F59000-Lille, France

(3) CHU Lille, Department of Biostatistics, F59000-Lille, France

(4) Univ. Lille, Lille University Hospital, ULR 2694 – METRICS: Evaluation des Technologies de santé et des Pratiques médicales, F-59000, Lille, France

(5) Department of Digestive Surgery and Transplantation, Lille University Medical Hospital, University of Lille Nord de France, F59000-Lille, France

**Name, postal and e-mail addresses of the author responsible for correspondence and to whom requests for reprints should be addressed:**

ROUSSELEAU DAMIEN [damien.rousseleau@chu-lille.fr](mailto:damien.rousseleau@chu-lille.fr)

Adress : Department of Anesthesiology and Critical Care, Hospital Claude Huriez, Lille University Hospital, F59000-Lille, France

ORCID: 0000-0002-9118-8840

**Supplementary Materials - Index**

| **Supplementary Methods** | |  |
| --- | --- | --- |
| Anesthetic Management | | *page 2* |
| Full Statistical Analysis section | | *page 2* |
| **Supplementary Figures and Tables** |  |  |
| Table S1 | *page 3* |  |

**Supplementary Methods**

**Anesthetic Management**

All patients received sublingual midazolam premedication (0.05–0.1 mg/kg) approximately 1 hour before surgery. Induction was with propofol (2–4 mg/kg), sufentanil (0.2–0.3 µg/kg), and atracurium (0.5 mg/kg). General anaesthesia was maintained with target-controlled propofol infusion (BIS 40–60) and additional sufentanil boluses (5µg) for hemodynamic changes. Atracurium (10 mg) was administered to maintain deep muscle relaxation. Ondansetron (4 mg) was given postoperatively for nausea and vomiting. Systematic analgesia was provided 30 mn before the end of the surgery with administration of 1g IV paracetamol and 20 mg IV nefopam.

**Full Statistical Analysis section**

It was planned to randomise 186 patients (62 per arm) to achieve 80% power to demonstrate the superiority of postoperative CWI or preoperative ITM combined with IV analgesic (two experimental arms) over IV analgesic alone (one control arm) in reducing 24-hour morphine consumption (primary outcome). The sample size was calculated using a two-sided t-test at the 0.025 significance level (regarding the Bonferroni correction to take into account the two comparisons between experimental arms and control arm), assuming a 30% reduction in 24-hour morphine consumption in each experimental arm compared to the control arm, with a mean 24-hour morphine consumption of 40 ± 20 mg in the control arm. An attrition rate of 10% was considered.

All analyses were performed on all randomised patients in their original group of randomisation whatever any protocol deviations (according to an intention-to-treat basis). A per-protocol sensitivity analysis was conducted for the primary endpoint after excluding 6 patients who did not receive allocated intervention (figure 1).

Categorical variables were expressed as numbers (percentages), and quantitative variables as means (standard deviation, SD) for normally distributed data or medians (interquartile range, IQR) otherwise. Normality was assessed using histograms and the Shapiro-Wilk test.

Cumulative morphine consumption at 24 (primary endpoint), 48, and 72 hours postoperatively in each experimental arm (CWI and ITM) was compared to the control arm (IV analgesic alone) using one-way analysis of variance (ANOVA), with log-transformation (values+1) applied to meet normality assumptions. Effect sizes were expressed as mean differences in log-transformation values with 95% confidence intervals (CIs). Secondary quantitative outcomes (length of hospital stay, length of ICU stay, ondansetron consummation at 72 hours, time to first gas, time to first bowel movement, time to resume normal feeding, consumption of supplementary analgesics at 72 hours, and tramadol consumption) were compared between each experimental and control arm using Dunn’s test (post-hoc Kruskal-Wallis^1^); effect sizes were expressed as standardised means differences calculated on rank-transformed data^2^. Binary outcomes (operative nausea and vomiting) were compared using chi-square tests, and relative risk was calculated as effect sizes; no statistical comparisons were made for pruritus and respiratory depression given the low number of events (0 and 2 for all group pooled together, respectively. Finally, the pain intensity at rest and on coughing or mobilisation assessed at 6 post-operative times (3, 6, 12, 24, 48 and 72 hours) were compared between each experimental and control arms using linear mixed models (an unstructured covariance pattern model to account for the correlation between repeated measures within the same patients) with baseline values, times, analgesia groups and interaction between times and analgesia groups as fixed effects. Post-hoc comparisons and mean differences between experimental and control arms at each time were done using linear contrasts.

All statistical tests were two-sided, and p-value < 0.025 was considered statistically significant after applying Bonferroni correction to account for the comparisons of the two experimental arm with control arm. All confidence intervals (CIs) were two-sided at 95% confidence level, and should not be used for hypothesis testing. Data were analysed using SAS (release 9.4) and R (release 3.6.2).

1. Dunn OJ. Multiple Comparisons Using Rank Sums. Technometrics. 1964;6:241‑52.

2. Yang D, Dalton J. Unified Approach to Measuring the Effect Size Between Two Groups Using SAS. SAS Global Forum 2012. 2012.

**Supplementary Table**

**Supplemental table S1. Comparison of total morphine dose over the 72 hours postoperatively between three analgesic groups after adjustment for the complexity of resection (an unplanned supportive analysis)**

|  | **Analgesic groups** | | | **Effect size** |  | **Effect size** |  |
| --- | --- | --- | --- | --- | --- | --- | --- |
| **Cumulative morphine dose**  **(mg)** | **IV analgesic (n=62)** | **CWI**  **(n=62)** | **ITM**  **(n=62)** | **IV analgesic vs CWI** | **P1^1^** | **IV analgesic vs ITM** | **P2^1^** |
| **At 24h (primary endpoint)** | 2.53 (2.28 to 2.79) | 2.54 (2.28 to 2.79) | 2.00 (1.75 to 2.27) | -0.003 (-0.36 to 0.36) | 0.99 | 0.53 (0.16 to 0.89) | 0.004* |
| **At 48h** | 2.93 (2.68 to 3.19) | 2.92 (2.67 to 3.16) | 2.75 (2.50 to 3.01) | 0.01 (-0.34 to 0.37) | 0.94 | 0.18 (-0.18 to 0.54) | 0.33 |
| **At 72h** | 3.21 (2.94 to 3.48) | 3.18 (2.92 to 3.44) | 3.23 (2.96 to 3.49) | 0.03 (-0.34 to 0.41) | 0.87 | -0.02 (-0.39 to 0.36) | 0.92 |

*Values are complexity-resection adjusted mean (95%CI) of logarithm transformation +1 values.*

*Abbreviations: CI, confidence interval; CWI, Continuous wound infusion; IQR, Interquartile range ; ITM, intrathecal morphine; IV analgesic, Intravenous analgesic. P1 and P2 indicated the p-values for comparison with control analgesia group. * statistically significant at two-sided significance level of 0.025 (Bonferroni adjusted significance level for the two experimental arms comparisons with control arm). The widths of the CIs were not adjusted for multiple comparisons and should not be used for hypothesis testing. Effect sizes were the complexity-resection adjusted mean difference (95%CI) for IV analgesic vs CWI or for IV analgesic vs ITM in logarithm (cumulative morphine dose+1) values.*
